# Platelet Inhibition Prevents NLRP3 Inflammasome Activation and Sepsis-Induced Kidney Injury

**DOI:** 10.3390/ijms221910330

**Published:** 2021-09-25

**Authors:** Marivee Borges-Rodriguez, Corbin A. Shields, Olivia K. Travis, Robert W. Tramel, Cedar H. Baik, Chelsea A. Giachelli, Geilda A. Tardo, Jan Michael Williams, Denise C. Cornelius

**Affiliations:** 1 Departments of Pediatrics, University of Mississippi Medical Center, Jackson, MS 39212, USA; marivee.borges@cchmc.org; 2 Emergency Medicine, University of Mississippi Medical Center, Jackson, MS 39212, USA; cashields@umc.edu (C.A.S.); rtramel@umc.edu (R.W.T.); cbaik@umc.edu (C.H.B.); cgiachelli@umc.edu (C.A.G.); gtardo@umc.edu (G.A.T.); 3 Cardiovascular-Renal Research Center, University of Mississippi Medical Center, Jackson, MS 39212, USA; olivia.travis@stjude.org (O.K.T.); jmwilliams5@umc.edu (J.M.W.); 4 Pharmacology & Toxicology, University of Mississippi Medical Center, Jackson, MS 39212, USA

**Keywords:** sepsis, NLRP3, platelets, Clopidogrel, multi-organ injury, endothelial activation, inflammation

## Abstract

Platelets, cellular mediators of thrombosis, are activated during sepsis and are increasingly recognized as mediators of the immune response. Platelet activation is significantly increased in sepsis patients compared to ICU control patients. Despite this correlation, the role of activated platelets in contributing to sepsis pathophysiology remains unclear. We previously demonstrated NOD-like receptor protein 3 inflammasome (NLRP3) inflammasome activation in sepsis-induced platelets from cecal-ligation puncture (CLP) rats. Activated platelets were associated with increased pulmonary edema and glomerular injury in CLP vs. SHAM controls. In this study, we investigated whether inhibition of platelet activation would attenuate NLRP3 activation and renal and pulmonary injury in response to CLP. CLP was performed in male and female Sprague Dawley (SD) rats (*n* = 10/group) to induce abdominal sepsis and SHAM rats served as controls. A subset of CLP animals was treated with Clopidogrel (10 mg/kg/day, CLP + CLOP) to inhibit platelet activation. At 72 h post-CLP, platelet activation and NLRP3 inflammasome assembly were evaluated, IL-1β and IL-18 were measured in plasma, and tissues, renal and pulmonary pathology, and renal function were assessed. Activated platelets were 7.8 ± 3.6% in Sham, 22 ± 6% in CLP and significantly decreased to 14.5 ± 0.6% in CLP + CLOP (*n* = 8–10/group, *p* < 0.05). NLRP3 inflammasome assembly was inhibited in platelets of CLP + CLOP animals vs. CLP. Significant increases in plasma and kidney IL-1β and IL-18 in response to CLP were decreased with Clopidogrel treatment. Renal injury, but not lung histology or renal function was improved in CLP + CLOP vs. CLP. These data provide evidence that activated platelets may contribute to sepsis-induced renal injury, possibly via NLRP3 activation in platelets. Platelets may be a therapeutic target to decrease renal injury in septic patients.

## 1. Introduction

Sepsis is a life-threatening dysregulated host inflammatory response to an infectious agent [1] and one of the leading causes of death in the world. At least 1.7 million adults in America develop sepsis and nearly 270,000 Americans die as a result of sepsis yearly [2]. Sepsis leads to multi-organ dysfunction including acute lung injury (ALI) [3], acute kidney injury (AKI) [4,5], hematologic and central nervous system dysfunction, amongst others. Increasing number of organs involved in sepsis is associated with increased mortality [6]. Current treatment modalities focus on supportive care; however, mortality remains high. It has been reported that approximately 35 to 50% of in hospital deaths are due to sepsis [7]. It is crucial to identify a therapeutic target that can improve mortality due to sepsis.

Platelets have been recognized as having a role in immunity [8,9], besides their role in hemostasis and thrombosis. Thrombocytopenia is frequently seen in sepsis and is thought to occur due to platelet activation and consumption; however, its role in the disease process remains unclear. Thrombocytopenia is associated with development of (multi-organ failure) MOF and increased 90 day mortality [10,11,12,13,14]. Platelets may be an integral part of the inflammatory response to sepsis. A potential mechanism by which platelets may contribute to multi-organ injury is through activation of the NOD-like receptor protein 3 inflammasome (NLRP3). NLRP3 is a cytosolic immune signaling receptor which leads to caspase-1 mediated cleavage and activation of IL-1B and IL-18 and has been implicated in the pathogenesis of inflammatory disease processes like asthma [15], Parkinson’s [16], inflammatory bowel disease [17] and sepsis [18]. Inhibition of NLRP3 inflammasome assembly has been shown to be protective against sepsis induced organ injury [19,20,21,22,23]. We have previously demonstrated that NLRP3 inflammasome is activated in platelets in response to cecal ligation-puncture (CLP) and is associated with multi-organ injury in response to polymicrobial sepsis in a 72 h CLP rat model. In the present study, we hypothesized that blocking platelet activation with Clopidogrel (CLOP) would decrease NLRP3 inflammasome activation, renal and pulmonary injury in response to polymicrobial sepsis.

## 2. Results

### 2.1. Plate Activation and NLRP3 Inflammasome Assembly

**Figure 1:**
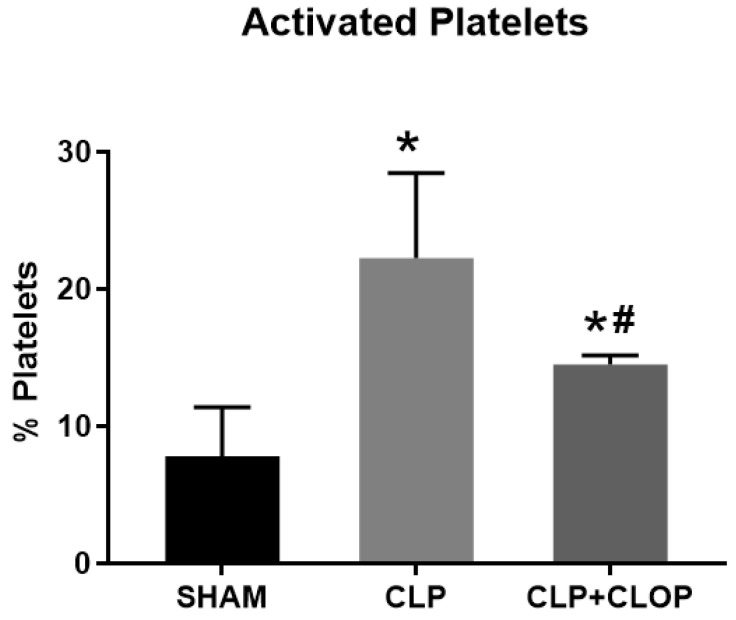
Platelet Activation. Platelet activation was measured by flow cytometry. Platelets were isolated form Sham, Cecal ligation and puncture (CLP) and CLP + Clopidogrel (CLP + CLOP). Activated platelets were identified as CD41^+^/CD62P^+^. * *p* < 0.05 vs. Sham; # *p* > 0.05 vs. CLP.

**Figure 2:**
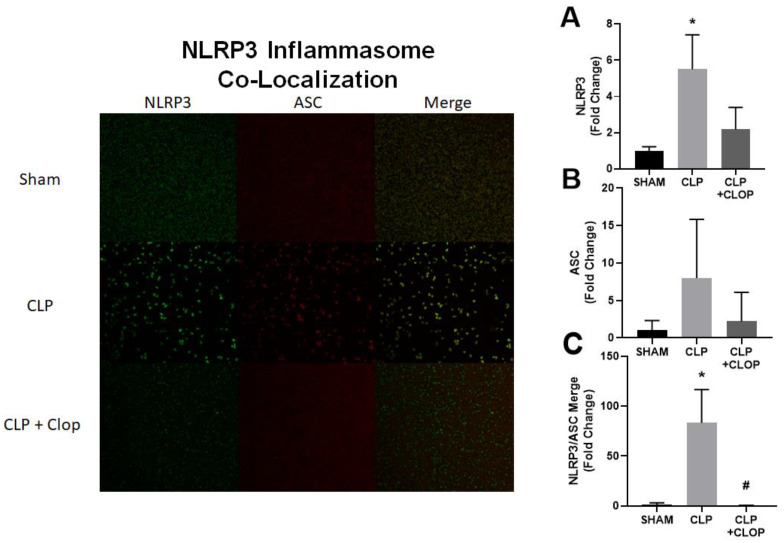
NLRP3 Inflammasome. (**A**–**C**) NLRP3 inflammasome activation was evaluated by visualizing the co-localization of NLRP3 and apoptosis-associated speck-like protein (ASC) using immunocytochemistry. * *p* < 0.05 vs. Sham, # *p* < 0.05 vs. CLP.

Using flow cytometry, we determined percent of platelet activation among groups. We found that platelet activation was increased in CLP rats by 22 ± 6%, when compared to SHAM 7.8 ± 3.6% (*p* < 0.05), and attenuated in CLP + CLOP rats by 14.5 ± 0.6% (*p* < 0.05, Figure 1). NLRP3 inflammasome assembly in platelets was assessed by visualizing co-localization of NLRP3 with apoptosis-associated speck-like protein (ASC) using immunocytochemistry (Figure 2). Co-localization of inflammasome components in CLP platelets was 83 times greater compared with Sham platelets (Figure 2C). Furthermore, assembly of the inflammasome was decreased in platelets of CLP + CLOP when compared with CLP.

### 2.2. Inflammasome-Associated Cytokines

**Figure 3:**
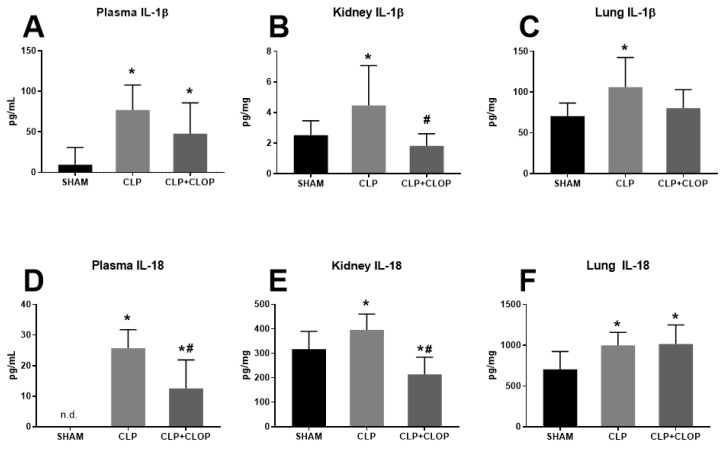
Inflammasome-associated cytokines. Plasma (**A**,**D**), kidney (**B**,**E**), and lung (**C**,**F**) IL-1*β* and IL-18 were measured in Sham, CLP, and CLP rats treated with Clopidogrel (*n* = 12/group) via ELISA. * *p* < 0.05 vs. Sham, # *p* < 0.05 vs. CLP, n.d. = not detected.

NLRP3 activation results in release of pro-inflammatory cytokines IL-1β and IL-18 into their active forms. We measured both cytokines in plasma, lung, and kidney homogenates of SHAM, CLP and CLP + CLOP via ELISA. Plasma IL-1β significantly increased from 9 ± 21 pg/mL in Sham to 77 ± 31 pg/mL in CLP (*p* < 0.05, Figure 3A). Administration of Clopidogrel showed a decrease in plasma IL-1β 48 ± 38 pg/mL; however, the decrease was not statistically significant when compared to CLP (*p* < 0.05 vs. Sham). Similarly, renal IL-1β was elevated in CLP 4 ± 3 pg/mg, when compared to Sham 2.5 ± 0.9 pg/mg (*p* < 0.05, Figure 3B). Treatment with Clopidogrel significantly decreased renal IL-1β to 1.8 ± 0.8 pg/mg (*p* < 0.05). In the lungs, IL-1β was significantly increased from 70 ± 16 pg/mg in Sham to 106 ± 36 pg/mg in CLP (*p* < 0.05) and was not significantly different in Clopidogrel treated animals (80 ± 23 pg/mg, Figure 3C).

Plasma IL-18 was not detectable in SHAM rats; however, average plasma IL-18 was 26 ± 6 pg/mL in CLP. Importantly, administration of Clopidogrel resulted in lower levels of plasma IL-18 (12 ± 9 pg/mL; *p* < 0.05, Figure 3D). Similarly, renal IL-18 was elevated in CLP (396 ± 65 pg/mg) compared to Sham (317 ± 73 pg/mg; *p* < 0.05). Treatment with Clopidogrel decreased renal IL-18 to 214 ± 70 pg/mg (*p* < 0.05, Figure 3E). In the lungs, IL-18 was significantly increased from 704 ± 219 pg/mg in Sham to 999 ± 158 pg/mg in CLP (*p* < 0.05) and remained elevated in Clopidogrel treated animals 1014 ± 235 pg/mg (*p* < 0.05 vs. Sham, Figure 3F).

### 2.3. Endothelial Activation

**Figure 4:**
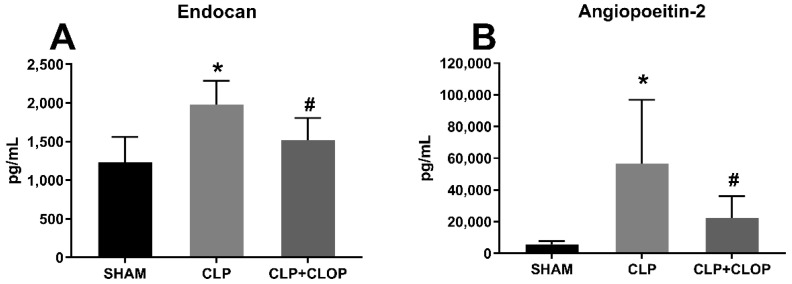
Endothelial activation. Plasma levels of endocan (*n* = 10/group) (**A**) and angiopoietin-2 (*n* = 7–8/group) (**B**), soluble markers of endothelial activation were measured in Sham, CLP and CLP + CLOP via ELISA. * *p* < 0.05 vs. Sham, # *p* < 0.05 vs. CLP.

We measured plasma levels of angiopoietin-2 and endocan as markers of endothelial activation in Sham, CLP and CLP + CLOP groups via ELISA. Plasma endocan was significantly increased from 1234 ± 327 pg/mL in Sham to 1978 ± 306 pg/mL in CLP (*p* < 0.05 vs. Sham). Endocan was significantly reduced to 1521 ± 283 pg/mL in Clopidogrel treated CLP rats (*p* < 0.05 vs. CLP, Figure 4A). Similar results were found for angiopoietin-2. Angiopoietin-2 was significantly increased from 5603 ± 2142 pg/mL in Sham to 56,767 ± 40,000 pg/mL in CLP (*p* < 0.05 vs. Sham) and was significantly decreased in Clopidogrel treated rats to 22,486 ± 13,616 pg/mL (*p* < 0.05 vs. CLP, Figure 4B).

### 2.4. Lung Injury

**Figure 5:**
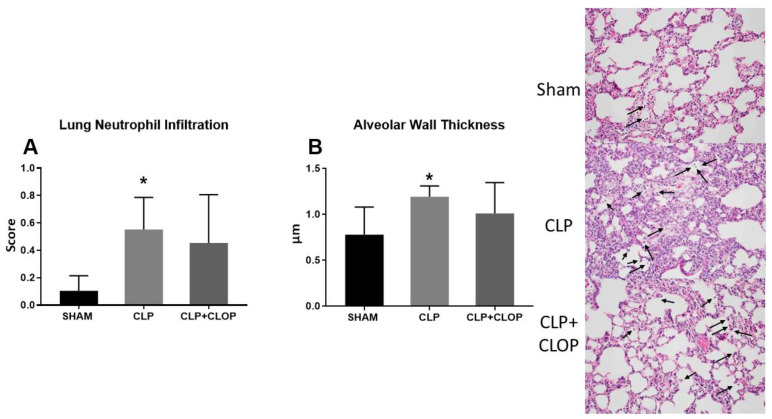
Lung injury. Neutrophil Infiltration (**A**) and alveolar wall thickness (**B**) into pulmonary tissues were assessed in Sham, CLP, and CLP + CLOP. H&E staining was performed on paraffin-embedded lungs from Sham, CLP, and CLP + CLOP rats (Representative images) (*n* = 6/group). Black arrows indicate neutrophil infiltration. * *p* < 0.05 vs. Sham.

Histological analysis of lungs from each group demonstrated a significant increase in neutrophil infiltration with an average score of 0.6 ± 0.2 in CLP rats compared to a score of 0.10 ± 0.10 in Sham rats (*p* < 0.05). We also observed that Clopidogrel administration to CLP rats did not alter neutrophil infiltration (score 0.5 ± 0.4, Figure 5A). Similar results were found in alveolar wall thickness with a significant increase in CLP with an average thickness of 1.2 ± 0.1 μm compared to 0.8 ± 0.3 μm in Sham rats (*p* < 0.05). Clopidogrel administration to CLP rats did not improve alveolar wall thickness (Figure 5B).

### 2.5. Kidney Injury

**Figure 6:**
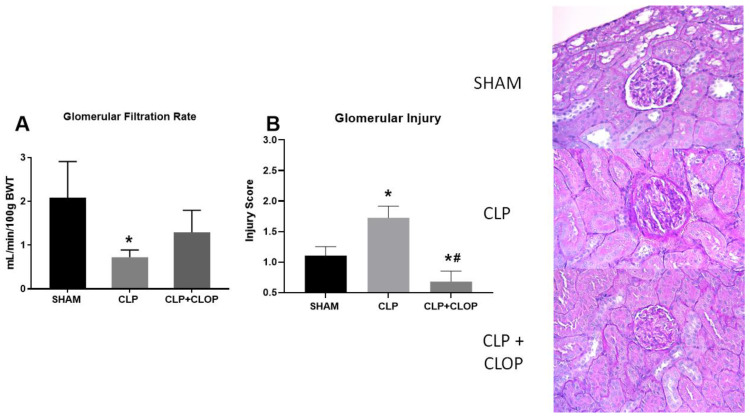
Renal function and injury. Glomerular filtration rate was assessed via FITC sinistrin clearance in Sham, CLP, and CLP + CLOP rats (**A**; *n* = 6). Periodic acid Schiff (PAS) staining was performed on paraffin-embedded kidneys from Sham, CLP, and CLP + CLOP rats (representative images). Glomerular injury (**B**; *n* = 6/group) was scored from PAS-stained sections. * *p* < 0.05 vs. Sham, # *p* < 0.05 vs. CLP.

As we have previously published, GFR significantly decreased from 2.1 ± 0.8 mL/min/100 g bwt in Sham rats to 0.7 ± 0.2 mL/min/100 g bwt in CLP rats (*p* < 0.05). Administration of Clopidogrel showed improved GFR to 1.3 ± 0.5 mL/min/100 g bwt in comparison to CLP, however not statistically significant (Figure 6A). Histological analysis demonstrated increased glomerular damage in CLP group compared Sham. Clopidogrel treatment significantly improved glomerular injury in CLP animals (Figure 6B).

## 3. Discussion

Sepsis is a dysregulated immune response to an infectious agent, often associated with multi-organ failure and known to have a high mortality. There is a need for the identification of novel therapy that may modulate inflammatory response and decrease mortality. Platelets have been postulated to have a role in immunomodulation. Naturally, the coagulation cascade and platelet activation has been a prominent focus of investigation. Administration of protein C, an anticoagulant endogenous enzyme, has been associated with improved survival in patients with severe sepsis [24]. In Japan, sepsis guidelines include the use of timed antithrombin administration in septic patients with DIC and have shown improved outcomes [25]. This suggest that inhibiting platelet activation may improve outcomes in sepsis. The inflammatory response during sepsis is definitely complex and may involve more than one pathway. We previously published that platelets from the CLP polymicrobial sepsis model have increased platelet NLRP3 assembly/activation and was associated with lung and renal injury [26]. In this study, we set out to determine the role of platelet inhibition in the inflammatory response in the same model using the P2Y12 inhibitor, Clopidogrel.

As previously published, platelet activation was increased in response to polymicrobial sepsis in the CLP model when compared with Sham rats. We found that administration of Clopidogrel decreased platelet activation in CLP rats and interestingly, a decrease in assembly of NLRP3 inflammasome in platelets was also observed. We also found a trend in decreasing circulating IL-1B and IL-18 in the plasma of Clopidogrel treated animals when compared to CLP group. In a previously published study, we reported that direct inhibition of NLRP3 inflammasome with MCC950 resulted in decreased IL-1B and IL-18 in plasma [27]. This suggests that inhibition of platelet activation with Clopidogrel and the decrease in inflammatory cytokines in this sepsis model is mediated, in part, by NLRP3 mechanisms. Therefore, further studies need to be performed to more clearly define the inflammatory pathways mediated by NLRP3 in platelets.

Multi-organ failure (MOF) occurs frequently in sepsis. Previously published studies reported that in mice, the CLP model caused significant pulmonary damage; and depletion of platelets reduced CLP-induced edema and neutrophil recruitment in the bronchoalveolar space by >60% [28]. We have previously published that increased platelet activation was associated with multiple organ dysfunction and injury in the CLP rat model [26]. Our data shows significant decrease in IL-1β and IL-18 in the kidneys of clopidogrel treated animals. The combination of acute renal failure and sepsis is associated with 70 percent mortality, as compared with 45 percent mortality among patients with acute renal failure alone [29]. Glomerular filtration rate (GFR) is an indicator of kidney function and declines with increased renal damage. Clopidogrel treatment improved renal injury in CLP rats, which suggests that platelet activation may play a role in the development of sepsis-induced AKI.

Consistent with its role in causing MOF, development of ALI and acute respiratory distress syndrome (ARDS) is commonplace in sepsis patients and often results in the need for mechanical ventilation in this patient population. Evidence suggests that platelet activation plays an important role in developing of MOF in septic patients [8,30]. Previous studies have supported this by displaying reduced neutrophil infiltration in the lungs of CLP-induced septic mice after platelet depletion [28]. Furthermore, Liverani et al. reported that mice pretreated with Clopidogrel were resistant to sepsis-induced ALI [31]. However, the current study was unable to show any significant evidence of decreasing IL-1β, neutrophil infiltration, or alveolar wall thickness in lungs. This may be due to use of a different animal species, dose of Clopidogrel, and other differences in experimental design.

Capillary leakage is a prominent feature of sepsis. Proteins such as the vascular growth factor angiopoetin-2 are known to promote inflammation, endothelial dysfunction and are associated with increased hospital mortality in patients with sepsis [32]. Endocan is another marker of endothelial cell dysfunction and has been related to the severity of illness and the outcome of septic patients [33]. We also found that these markers of endothelial activation and dysfunction were elevated in CLP model, and decreased in the Clopidogrel treatment group. Similarly, we observed a slight, but insignificant increase in GFR in the Clopidogrel treated group. This suggests that platelet activation has some modulatory effect in the inflammatory response to sepsis.

In this study, the inhibition of platelet activation did not translate to significant improvements in organ function. It is possible that the chosen dose of 10 mg/kg/day was sufficient to attenuate platelet activation, NLRP3 activation, cytokines, and markers of endothelial activation, but not translate to an attenuation of lung injury or improvement in renal function. Liverani, et al. previously used an initial dose of 30/mg/kg/day in wild-type and P2Y12 knock-out mice, which did translate to inhibition of sepsis-induced lung injury [31]. In the same study, Clopidogrel was administered prior to induction of sepsis. This major difference may explain the lack of significant improvements in lung or renal function observed in our study.

While the current study suggests a role for platelet activation in contributing to inflammation and endothelial activation in response to sepsis, there are limitations that should be noted. First, while markers of endothelial activation were measured, additional experiments to assess endothelial function are warranted. Moreover, the contribution of other platelet-derived factors was not controlled for in this study. Thus, there are still unanswered questions regarding how platelets contribute to sepsis induced pathology and organ failure.

The role of Clopidogrel in reducing mortality in sepsis has been controversial with reports of decreasing incidence of ARDS and mechanical ventilation, but no decrease in mortality [34]. Further studies need to be performed to determine the exact mechanism by which platelets contribute to organ injury and dysfunction in sepsis. This study supports further exploration of platelets as a therapeutic target in septic patients.

## 4. Materials and Methods

### 4.1. Animals

Female and male Sprague Dawley rats purchased from Envigo (Indiandapolis, IN, USA) were used. All experimental procedures executed in this study were in accordance with the National Institutes of Health guidelines for use and care of animals. All protocols were approved by the Institutional Animal Care and Use Committee at the University of Mississippi Medical Center. The care and handling of the animals were in accordance with the National Institutes of Health guidelines for ethical animal treatment.

### 4.2. Cecal Ligation and Puncture

All of our in vivo experiments were performed in 12- to 13-week-old male and female rats weighing approximately 225–325 g. The animals were randomly divided into two groups: sham operation group (SHAM, *n* = 10) and cecal ligation puncture group (CLP, *n* = 10). Under isoflurane anesthesia the CLP surgery was performed on a subset of rats to induce abdominal polymicrobial sepsis as we have previously described [26,27]. Preheated saline (20 mL/kg, 37 °C) was subcutaneously injected immediately after the operation for resuscitation. Sham-operated animals underwent the same surgical procedure without cecum ligation or puncture. The rats were placed back into their cages after surgery, and food and water were provided ad libitum. At 24 h post-CLP, the prior incision was reopened and the necrotic cecum was carefully excised. The abdominal cavity was washed with 10 mL of warm sterile saline solution. The abdominal incision was again closed in layers. Animals were administered a broad-spectrum antibiotic daily (Naxcel 5 mg/kg) and analgesia achieved with buprenorphine SR (1.2 mg/kg) over the 72 h experimental period. Platelet aggregation was blocked by daily administration of Clopidogrel (10 mg/kg/day) for 3 days in a subset of CLP rats (CLP + CLOP; *n* = 12) via esophageal gavage for 3 days. At the end of the protocol, the animals were sacrificed under deep anesthesia. Blood and tissues were collected for further analysis.

### 4.3. Platelet Isolation

Arterial blood was drawn into acid-citrate-dextrose vacutainer tubes and centrifuged at 200× *g* for 20 min to obtain platelet-rich plasma. Platelet-rich plasma (PRP) was mixed with an equal volume of HEP buffer (140 mmol/L NaCl, 2.7 mmol/L KCl, 3.8 mmol/L HEPES, 5 mmol/L EGTA, pH 7.4) containing 1 μM PGE1. This mixture was then centrifuged at 100× *g* for 20 min to pellet remaining RBC and WBC in which 40 μL of the supernatant were used to count platelets. The PRP was then centrifuged at 800× *g* for 20 min, the supernatant was discarded and the pellet was resuspended in Tyrode’s buffer (134 mmol/L NaCl, 12 mmol/L NaHCO_3_, 2.9 mmol/L KCl, 0.34 mmol/L Na_2_HPO_4_, 1 mmol/L MgCl_2_, 10 mmol/L HEPES) containing 5 mmol/L glucose and 3 mg/mL BSA.

### 4.4. Flow Cytometry

Freshly isolated platelets (10^6^) were resuspended in 50 µL of Tyrode’s buffer. Platelets were incubated (RT for 30 min) with fluorescein isothocyanate (FITC) -conjugated anti-mouse CD41 (Clone MWReg30, BioLegend, San Diego, CA, USA) and Allophycocyanin (APC) conjugated anti-mouse/rat CD62P (Clone RMP-1, Biolegend). A minimum of 10,000 events per gate were acquired using a MACsQuant Analyzer 10 (Miltenyi Biotec, Auburn, CA, USA) and analyzed using FlowLogic software (Innovai, Sydney, Australia). Platelets were distinguished by specific binding of anti-CD41 and characteristic forward and side scattering. Platelets staining positive for CD41 and CD62P were designated as activated platelets.

### 4.5. Confocal and Light Microscopy

Platelets (10^7^) from Sham or CLP rats or stimulated with or without LPS were allowed to adhere to permanox slides (Lab-Tek, Thermo Fisher Scientific, Waltham, MA, USA). Adhered cells were fixed in 10% formalin for 30 min, washed 3Xs with PBS for 10 min at RT, and permeabilized with ice cold 100% methanol for 10 min. The slides were blocked in PBS with 10% goat and rabbit serum for 1 h, washed and incubated with rabbit anti-NLRP3 antibody (NBP2-12446, Novus Biologicals, Littleton, CO, USA) for 2 h at RT. Slides were then washed and incubated with anti-rabbit Alexa Fluor 488 (A11070, Thermo Fisher Scientific) for 1 h at RT. The slides were washed again and incubated with Alexa Fluor 647-conjugated rabbit anti-ASC antibody (NBP1-78977AF647, Novus Biologicals) for 1 h at room temperature. Controls were processed identically, except for omission of the primary antibodies. Preparations were mounted in Cytoseal 60 (Thermo Fisher Scientific) and analyzed on a Nikon C1 (Nikon Inc., Melville, NY, USA) confocal scanning microscope. The images were captured using a Nikon Eclipse 55i microscope equipped with a Nikon DS-Fi1 color camera (Nikon) and analyzed using NIS-Elements D 3.0 software.

### 4.6. Quantification of Biomarkers of Endothelial Activation and Cytokines

Plasma levels of Angiopoietin-2 (MANG20, R&D Systems, Minneapolis, MN, USA) and Endocan-1 (MBS039900, MyBioSource, San Diego, CA, USA) were measured as markers of endothelial dysfunction by ELISA according to the manufacturer’s protocol. Plasma, kidney, and lung IL-1β (RLB00, R&D Systems) and IL-18 (ab213909, Abcam, Boston, MA, USA) were quantified by ELISA according to the manufacturer’s protocol. Kidneys and lung lysates were prepared with the BioPlex Cell Lysis Kit (171304011, BioRad, Hercules, CA, USA) according to the manufacturer’s protocol.

### 4.7. Assessment of Lung Injury

Histological evaluation was performed on lungs via hematoxylin and eosin (H&E) staining. A lung lobe was formalin-fixed. The tissue was subsequently paraffin-embedded and 5-µm sections were stained with (H&E). Sections were evaluated for neutrophil infiltration into the alveolar space. Five sections were scored in a blinded fashion on a scale of 0–2 with 0 representing o neutrophils in the alveolar space, 1 representing 1–5 neutrophils in the alveolar space, and 2 representing >5 neutrophils in the alveolar space. Images were captured using a Nikon Eclipse 55i microscope equipped with a Nikon DS-Fi1 color camera (Nikon Inc.) at 40X using NIS-Elements D 3.0 software. Alveolar wall thickness was measured using NIS-Elements D 3.0 software. Data were normalized to sham values.

### 4.8. Assessment of Renal Glomerular Injury

Kidneys were collected, weighed, and fixed in a 10% buffered formalin solution. Paraffin sections (3 μm) were prepared and stained with Periodic acid-Schiff to assess the degree of glomerular injury on approximately 30 images per section, per rat. Thirty glomeruli per section were scored in a blinded fashion on a 0–4 scale with 0 representing a normal glomerulus, 1 representing a 25% loss, 2 representing a 50% loss, 3 representing a 75% loss, and 4 representing >75% loss of capillaries in the tuft. Images were captured using the same microscope, camera, and software as mentioned previously.

### 4.9. Measurement of Renal Function

During the CLP procedure, a catheter was inserted into the jugular vein and exteriorized subcutaneously at the back of the neck. The catheter was flushed with heparinized saline each day until the end of the three-day protocol. On the last day of the protocol, rats underwent brief isoflurane anesthesia in order to assemble the NIC-Kidney device (MediBeacon; Mannheim, Germany) made of two light emitting diodes that excite FITC-sinistrin at 480 nm, a photodiode that emits light at 531 nm, a microprocessor, and a battery. This device was attached to the back of the rat using a double-sided adhesive patch (MediBeacon) and secured with a rodent jacket to a shaved region on the back of the rat. Rats were allowed to recover in separate cages for 15 min and a baseline measurement was recorded. After baseline measurements, a bolus injection of FITC-sinistrin (5 mg/100 g body weight; FTC-FS001, MediBeacon) was administered via the jugular vein followed by a bolus injection of sterile saline. During a two-hour period after the bolus injection, excretion kinetics of FITC-sinistrin were measured transcutaneously and were used to calculate elimination half-life (t_1/2_) of FITC-sinistrin using a one compartment model with the MDPLab evaluation software (MediBeacon) to calculate GFR from a validated empirically-derived conversion factor [35,36].

### 4.10. Statistical Analysis

Power analysis determine that an n of 10–12 animals/group would achieve a power (1-β) of 0.80 and a probability of Type 1 error (α) of 0.05 to detect a difference of at least 5–10% in platelet activation between the Sham and CLP rats. All data are expressed as mean ± SD. All data were checked to be consistent with Gaussian distribution by D’Agostino-Pearson normality test. Statistical analyses were performed with one-way ANOVA with Tukey’s multiple comparisons test as post hoc analysis. A value of *p* < 0.05 was considered statistically significant.

## Data Availability

All data in support of the reported results in presented in the manuscript and free available.
